# Characterizing core outcomes of responsible stewardship for human genomic data in the cloud

**DOI:** 10.1186/s12920-026-02382-x

**Published:** 2026-05-06

**Authors:** Vasiliki Rahimzadeh, Bronwyn Walsh, Heidi L. Rehm, Mildred Cho, Amy L. McGuire

**Affiliations:** 1 Center for Medical Ethics and Health Policy, Baylor College of Medicine, Houston, TX USA; 2 Center for Genomic Medicine, Massachusetts General Hospital, Boston, MA USA; 3 Program in Medical and Population Genetics at the Broad Institute of MIT and Harvard, Cambridge, MA USA; 4 Stanford Center for Biomedical Ethics, Stanford University, Stanford, CA USA

**Keywords:** Human genomic data, Data stewardship, Data sharing, Ethical data use, Cloud, Repository, Core outcome, Stewardship maturity

## Abstract

We present findings from a scoping review of the genomic data sharing literature used to inform a core outcomes set for responsible data stewardship in the cloud. Genomic and related health data stemming from government funded research have undergone mass migration to the cloud where they can be more securely stored, accessed, and analyzed within shared computing environments. While this migration reflects a shift in privacy and security infrastructure for cloud-based repositories as oversight becomes more globalized, it also necessitates new data stewardship responsibilities to align authorized data access with ethical data use. Responsible data stewardship refers to the ethical and secure management, use, and protection of data to ensure its accuracy, privacy, integrity, and appropriate access throughout its lifecycle. Practicing responsible data stewardship is therefore one axis by which repositories can demonstrate trustworthiness in their data access and management practices. We searched the cloud governance and data sharing literature indexed in Web of Science as well as Elicit using the scoping review approach by Arksey and O’Mally. A total of 46 articles met our inclusion criteria, to which we applied a deductive, thematic content analysis to generate relevant outcome domains as well as individual core outcomes of cloud-based data. Our qualitative synthesis resulted in 35 individual core outcomes organized under 9 core domains: transparency, authentication, auditing, accountability, security, sustainability, standardization, consent and compliance, and community engagement.

**Supplementary Information:**

The online version contains supplementary material available at 10.1186/s12920-026-02382-x.

## Background

More human genomic data are expected to be generated than Twitter, YouTube and the field of astronomy combined by as early as 2025 [1]. A late 2025 report estimated that global genomic data would surpass 40 billion gigabytes [2]. Furthermore, the NHGRI estimates that 2–40 billion more gigabytes of genomic data will be generated annually [3]. The secure storage, use, and exchange of these large human genomic and related health datasets therefore compel repositories, research institutions and funders to ensure compute power and adopt storage platforms that scale. Cloud technologies have quickly evolved [4–7] to become some of the most widely leveraged [8], and commercially available solutions to human genomics’ data infrastructure [9, 10] and training [11] problems. However, migrating these data to cloud platforms and the rise of cloud-based repositories has resurfaced previously identified ethical, legal, and social issues [12–14] and raised new policy challenges [9]. For example, many privacy and security systems were engineered for a pre-cloud world [15], when “seamless, borderless sharing and storage of data [16]” was not yet a computing reality.

In addition, existing data protection laws are jurisdiction-specific [17], which can create ambiguities regarding which data protection rules apply to whom, when, and where in the cloud because bona fide researchers can apply for access from virtually anywhere in the world with appropriate credentials [16, 18]. Not all cloud platforms are architected the same with implications for the security of their perimeters [19]. Private clouds are typically managed by one or more researchers, as part of a single laboratory or a research group. Public clouds, in contrast, often host large-scale data collections made accessible by research agencies such as the National Institutes of Health (NIH). Each type of cloud may enable adequate data storage and analysis for different projects and be cost efficient. However, as Heath and colleagues note, “Some concerns have been raised about using public clouds to house controlled-access genomics data—specifically, (1) the cost, (2) the security infrastructure, and (3) the wisdom in providing all of one’s research data to a company that might be acquired in the future or may decide to exit the business of cloud computing” [20].

Finally, public opinion surveys overwhelmingly suggest that people prefer knowing how and why their genomic data are used [21–28]. Because genomic data continues to be perceived to be highly sensitive, fear of discrimination, threats to privacy, and potential for misuse are frequently cited concerns about the prospect of widely sharing genomic data [29–31]. These trends are particularly pronounced among underrepresented racial and ethnic groups in genomics [29]. Moreover, studies have shown that prospective data contributors (i.e. individuals, data subjects, research participants) who provide their genomic and health related data to repositories for use in research) are most willing to share their data for university-based research, but least willing when for-profit companies and other third parties can access these data [23, 32]. It can therefore be challenging to know exactly how to respect consent restrictions for sharing data with industry when universities and research funders seek research partnerships [33] with commercial cloud service providers (CSP) to fulfill their data platform needs [34]. Some scholars even propose that control over research data clouds concentrated in high income countries creates new research “oligarchies” [35] that will continue to disadvantage science in the low- and middle- income countries.

Trusted data environments [36] offer possible technical solutions to better securing genomic data. However, operationalizing norms of data stewardship [37] is still needed to embed the rights and interests of data contributors as well as research equity into cloud architecture itself.

### Responsible data stewardship

In this paper, we adopt the following definition of data stewardship by Wendelborn and colleagues:

Data stewardship is the responsibility to optimise research data management and sharing to maximise the potential value of research data for the scientific community and the public and, at the same time, to respect the rights and legitimate interests of all other stakeholders concerned, such as data subjects, vulnerable groups or communities, data producing researchers, organisations in which the data stem from, research funders, scientific journals, and data repositories. [38]

Bartlett (2025) elaborates on the normative and practical purposes of data stewardship, arguing that it will “generate public trust by managing patients’ data rights” and “by sharing data for public benefit with increased institutional, legal, and ethical safeguards” [39]. Helpful frameworks such as FAIR (Findable, Accessible, Interoperable, Reusable) [40, 41], CARE (Collective Benefit, Authority to Control, Responsibility, Ethics) [42], and TRUST (Trust, Respect, Utility, Sustainability, Technology) broadly outline principles that relate concepts like access, data management, public good, ethics and stakeholder rights. These principles can, however, be difficult to implement in repository practice [43], and more so in emerging computing environments.

Recent international regulatory developments further underscore the growing need for updated frameworks. The 2025 European Health Data Space (EHDS) Regulation reflects a set of normative priorities closely aligned with data stewardship, including accountability, controlled access, and responsible data reuse [44]. For example, Article 73 stipulates that health data must be shared within secure processing environments [45]. As such, the EHDS not only operationalizes these principles through its governance architecture but also reinforces the broader significance in shaping trustworthy health data ecosystems that seek to balance individual rights, collective benefit, and responsible data use. Furthermore, the NIH recently released proposed changes to their data sharing policy (NOT-OD-26-023) [46]. These proposed changes underscore that genomic data stewardship by creating a unified, controlled-access framework that standardizes how sensitive data are governed, accessed, and stored across the data lifecycle. These changes shift the focus from simply sharing data to ensuring responsible stewardship that aligns with consent, privacy, and sustainable reuse.

However, the traditional data stewardship frameworks (FAIR, CARE, and TRUST), are ill-suited to the cloud because they assume locally controlled data management models. Cloud platforms, on the other hand, enable dynamic, distributed, and cross-border data use that complicates enforcement, oversight, and compliance [17, 47–49]. Thus, current governance approaches lack the specificity and adaptability needed to address the technical and regulatory complexities of cloud-based data stewardship [50].

Our paper centers on data stewardship, a concept that is related but distinct from a data trustee, which has received particular attention in European data stewardship and policy discussions. In this paper, we use data steward to refer to roles or entities concerned with enabling responsible data use through curation, quality assurance, access management, and governance practices. By contrast, data trustee typically denotes an entity with a stronger representative or fiduciary orientation, including responsibilities tied to protecting data subjects’ or stakeholders’ interests, as well as data protection and security. The two concepts overlap in their emphasis on trust, accountability, and governed data use, but they are not identical.

Moving repositories beyond regulatory compliance to responsible data stewardship requires finding better ways to align the values of diverse stakeholders with institutional practices for data release bespoke to the dynamic data sharing environment of the cloud [39, 51]. While compliance with applicable laws and regulations is fundamental, responsible stewardship of human genomic data is a normative goal that aims to calibrate data access with data use in line with diverse stakeholder interests (e.g. data producers, users, and contributors) [52]. Without an updated framework tailored to the cloud, repositories will be unable to gauge whether their practices conform to global perceptions of stewardship, nor will they recognize feasible areas for improvement when adapting to new computing environments.

What outcomes matter most for the purposes of data stewardship, and how should we accurately and consistently measure them over time? We aim to answer this question by adapting consensus-building methodology from clinical medicine to facilitate the generation of a core outcomes set (COS) [53] of responsible stewardship for human genomic data in the cloud. In the clinical trial context, a COS is a standardized set of outcomes that should be measured and reported in all studies for a specific health condition or intervention. The purpose of a COS is to improve consistency and relevance in outcome measurement to guide treatment decisions and disease management [53]. An analogous use case for a COS in the data stewardship context could be instructive for understanding what institutional policies and practices constitute responsible data stewardship and governance [54] when leveraging cloud technologies for human genomic data science, how the outcomes of responsible stewardship are consistently measured and transparently reported over time, as well as who should primarily act on stewardship performance evidence.

## Methods

In this paper we report findings from a scoping review of the genomic data sharing literature according to the approach proposed by Arksey and O’Malley [55]. The goal of the scoping review was to inform the development of an initial COS for responsible stewardship that could be tested in future work. We searched for articles indexed in Web of Science and PubMed and supplemented these conventional searches using Elicit. Elicit™ is a literature review assistant powered by generative AI and was trained on ~ 126 million records contained in Semantic Scholar at the time of writing [56]. Though still early in development and implementation, Elicit has been shown to be successful in comparative trials to support researchers in article search, screening and data extraction with human oversight [57]. Recent validation studies [58, 59] suggest that complementing, rather than replacing manual human review of search results and data extraction enhances sensitivity and specificity of literature findings; this review was conducted with these recommendations in mind.

We search Web of Science using “genomic data stewardship” or “genomic data management” or “genomic data governance” or “data ethic” or “data privacy;” “cloud computing” or “cloud infrastructure” or “cloud storage; and” “measurable outcomes” or “impact assessment” or “evaluation” or “metrics.”

Articles indexed in PubMed were found using the following search string: (((“genomic*“[All Fields] OR “genetic*“[All Fields]) AND (“stewardship“[All Fields] OR “stewardships“[All Fields])) OR “responsib*“[All Fields] OR “ethic*“[All Fields]) AND (“outcome“[All Fields] OR “outcomes“[All Fields]) AND ((“genome“[MeSH Terms] OR “genome“[All Fields] OR “genomes“[All Fields] OR “genome s“[All Fields] OR “genomically“[All Fields] OR “genomics“[MeSH Terms] OR “genomics“[All Fields] OR “genomic“[All Fields]) AND (“data basel“[Journal] OR “data“[All Fields])) AND (“cloud“[All Fields] OR “cloud s“[All Fields] OR “clouds“[All Fields]).

Search results in Elicit were generated in response to the following prompt: “What are the core outcomes of responsible stewardship of human genomic data managed in the cloud?” Elicit identified the most articles (*n* = 507), compared to Web of Science (*n* = 200) and PubMed (*n* = 7). This may be due to its broader and less curated set of sources, whereas PubMed and Web of Science prioritize precision, standardization, and vetted academic indexing [60–64].

**Fig. 1:**
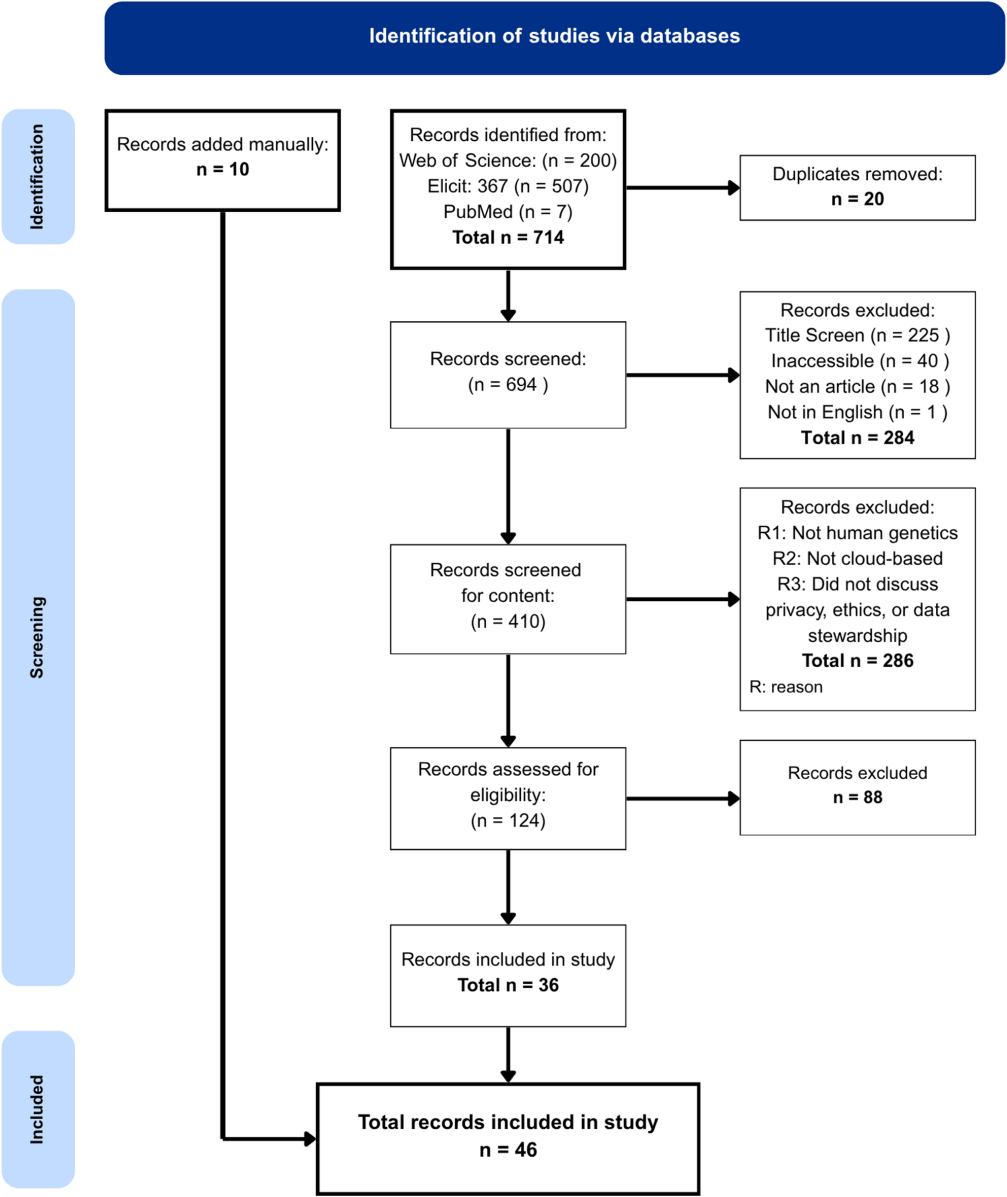
PRISMA Diagram of scoping review search for core outcomes of human genomic data stewardship in the cloud

A total of 714 articles were returned from our initial search across the three databases (Fig. 1). Articles were included if they:

- Published in English from 2011 to 2024 to ensure outcomes align with contemporary advances in cloud computing from the last 10 years.
- Discussed human genomic data management and access using cloud platforms, tools, or storage solutions.
- Explored issues of genomic data stewardship, including privacy and security, governance.
- Provided information about data uses.

After de-duplication, two independent reviewers conducted an abstract and title screen. Forty articles were inaccessible (i.e. inaccessible by our institution, unfindable on Google Scholar or our institutional library, Error 404 message), 18 results included other media not suitable for analysis (e.g. full-length books, seminar discussions, conference list of presentations, PhD theses), and 1 article lacked an English translation.

We excluded articles during our full text screen that did not discuss ethical, legal, or social issues related to data access and management. Furthermore, articles that discussed non-human genomics were excluded as well as articles that did not discuss the cloud, refer to the cloud, or that did not use cloud technologies for any part of data analysis, storage or sharing.

We also excluded articles that only discussed technical protocols (e.g. specific technological advances for coding DNA). Despite robust literature on cloud technology applications in human genomics, very few articles directly address the concept of “data stewardship,” including mechanisms for controlled access and governance of data use in accordance with regulatory and ethical requirements. Also given the diversity of literatures from which governance-related issues and themes were discussed, we manually added 10 relevant articles from reference lists of articles identified in the main literature search. Altogether, we included 46 works of literature in our analysis. List of 46 articles and corresponding publication information is available in Supplementary Materials 1.

We applied thematic content analysis using a priori codes developed based on prior qualitative work in this space [9, 65–67]. We deductively coded the literature based on three core phases of data stewardship responsibility and activity: pre-data collection, data storage and use, and secondary use and sharing. Coding the data by phase enabled us to capture the full scope of cloud-enabled data stewardship decisions at key timepoints across the data lifecycle. For example, we coded article content related to pre-data collection if the article discussed consent or methods of data procurement. Similarly, we coded article content under data storage and use if authors, for example, described consequences or institutional actions for data misuse or strategies for ensuring data integrity. Lastly, we coded article content under the secondary use and sharing phase if authors covered issues such as data deletion or research dissemination, among others.

The literature we synthesized often described in technical terms best practices and general recommendations for compliant data access, use and sharing. To translate the coded data by lifecycle stage into actionable outcomes, two analysts (VR and BW) generated outcome statements from protocol suggestions and technical recommendations articulated in the included literature. These recommendations frequently stemmed from genomic data science studies funded by federal research funding agencies and genomic research consortia, and aligned with existing data protection law/regulations [68, 69].

Following coding of outcome statements, we thematically categorized the outcome statements by theme, which comprised a set of core domains.

## Results

We identified core domains of genomic data stewardship across three principal phases in the data lifecycle, including (1) pre-data collection, (2) data storage and use, and (3) secondary use and sharing. Nine core domains were included in our final core outcomes set: Transparency, Accountability, Authentication, Auditing, Security, Sustainability, Standardization, Consent and Compliance, and Community Engagement, TA^3^S^3^C^2^. (pronounced “task”). The results are organized by core domain, and each begins with a definition that we derived based on our analysis, followed by a description of how the outcome domains were discussed in the data. Table 1 features the full list of the 35 individual core outcomes organized by domain. Reporting of our development process follows recommended guidelines from the core outcomes literature [70].

**Table 1:** Our team synthesized 9 core domains and 35 total outcomes from a scoping review of the cloud data sharing literature

Thematic Domain	Outcome	Literature
Transparency	Implications for future use of data are made clear to prospective data contributors	[71–73]
	Research findings and products made possible from data are disseminated to the public	[33, 72, 74]
	Data contributors are made aware of the rules, policies, and governance mechanisms of sharing their data.	[71, 73, 75–77]
Accountability	Instances of data misuse are investigated in a timely manner.	[78]
	Instances of unauthorized access are investigated in a timely manner.	[78]
	Actions taken to hold users accountable for violating terms of data access, use, and sharing are consistently enforced (e.g. penalties, loss of access privileges, additional training and intervention).	[72, 75, 79–81]
	Users adhere to applicable access, use, and sharing policies for data managed in cloud environments.	[33, 71–73, 82]
Authentication	Only researchers with bona fide institutional credentials and other authorized users are granted access to requested controlled-access datasets.	[49, 72, 79, 80, 82–84]
	Authentication processes for authorized data accessors are consistently applied.	[33, 78, 82, 85–89]
	Data should be screened for malware prior to being added to a repository	[71, 90]
	Researchers and other authorized users easily prove their identity using standardized identity providers	[71]
Auditing	Repository managers and institutional data custodians confirm that authorized data accessors use data only for approved purposes as indicated by an institutional review board (or research ethics committee), data access review committee, or other institutional ethics oversight body.	[71, 72, 79, 89]
	Information about users’ access to data held within controlled-access repositories are tracked and logged in a standard, secure way.	[33, 80, 82, 86, 89, 91]
	Institutions log and track data access decisions.	[71, 92]
	Repository managers and custodians monitor data access activities of all users in the cloud.	[49, 79, 82, 89, 90, 93–95]
Security	Data security protocols should be proportionate to the levels of protection required (ex: different levels of DAC oversight)	[33, 71, 74, 96, 97]
	Highest accepted industry standards (encryption, multi-factor authentication, differential privacy, etc.) are utilized to secure the data.	[49, 71, 72, 89, 90, 97–103]
	Individuals are notified of any data breach or violations of security conditions.	[78, 90, 95]
	Instances of data fabrication or inaccuracies are reported and investigated in a timely manner.	[33, 82, 86]
	Source data are not modified or tampered with by users.	[71, 82, 89, 90, 96, 104]
	Screens for malware show no tampering or spoofing of the datasets contained in the cloud	[82, 89]
Sustainability	Data are indexed such that researchers can find data quickly and efficiently.	[72, 78, 80, 85, 88, 90, 105, 106]
	Datasets are preserved in the cloud even after initial funding for the database or repository sunsets.	[96, 101]
	Cloud-based databases and repositories have adequate human and compute resources to functionally operate.	[33, 78, 81, 90, 91, 95, 99, 104]
Standardization	Terms of data use are standardized across platforms and cloud databases/repositories.	[78]
	The database/repository supports cloud federation.	[88, 89, 99, 106–110]
	Database/repository stores and shares data in standard file formats.	[88, 110–112]
Consent and Compliance	Databases/repositories contain data that have been properly de-identified and/or provide aggregate summary data according to applicable privacy rules/laws.	[75, 78, 82, 110, 113, 114]
	Consent for all contributed data is obtained and recorded in machine readable terms.	[71, 72, 104]
	Unauthorized re-identification of data contained in cloud databases/repositories is met with enforceable penalties and consequences as set out by the institution.	[72, 80, 82, 90]
	Institutions restrict the sharing of unnecessary data (data minimization) held in controlled-access databases/repositories beyond what is outlined in a data access request and needed to answer a research question.	[71, 73, 81, 82, 93, 98]
	Data are deleted from cloud databases/repositories, when possible, following the withdrawal of consent from data contributors or after the elapsed storage time specified at the time of consent.	[74, 80, 104]
	Data contributors maintain legal recourse if they are harmed or suffer damages as a result of data misuse or unauthorized access.	[78]
Community Engagement	Communities that adopt community consent practices for the access, use, and sharing of prospective and legacy datasets are appropriately consulted in data access decisions.	[72, 74]
	Researchers and institutions provide clear information about data use, including assurances of ethics oversight to protect contributor rights, particularly with an identifiable Indigenous community or vulnerable group.	[33, 73, 75, 114]

### Transparency

We defined transparency as *the practice of openly communicating policies*,* processes*,* and decisions related to the use*,* sharing*,* and governance of genomic data*,* fostering trust among participants and stakeholders*. Transparency is a foundational component of the ‘broad consent’ process- the agreement by participants to the storage and future unspecified use of their genomic data for research purposes [115]. This consent framework is typical of participation in genomic research during the data collection phase, and in dictating access and management decisions in the data use and sharing phases. While broad consent is widely recognized and supported in some jurisdictions, it remains a contested legal concept in others [116–118]. Transparency about future research use furthermore enables prospective data contributors to outline use restrictions, if any, and the types of research they expect their data to advance. Several articles also pointed to transparency as it relates to the scientific and social benefits derived from the contributed data, including making publications and other research outputs available to the public and including vulnerable populations in discussions about data use. This corroborates a prior global survey that found transparency about who benefits from genomic research is the most important indicator of trustworthiness for data sharing [119].

### Accountability

We defined accountability as the *obligation and mechanisms by which individuals*,* organizations*,* and systems are held responsible for the ethical*,* legal*,* and secure handling of genomic data*. Accountability in cloud-based environments often *involves both technical controls (e.g. encryption*,* access logs*,* identity management) and governance frameworks to ensure that those handling genomic data do so responsibly and in alignment with ethical and legal standards*. Accountability was primarily discussed at the data use and sharing phases, ensuring that misuse and unauthorized access are detected, investigated, and swiftly addressed through consistent enforcement. Applying penalties and conducting compliance audits were the most frequently supported mechanisms for identifying violations. Accountability correlated strongly with perceptions of contributor trust in having data more widely available on cloud platforms.

### Authentication

We defined authentication as *the process of verifying the identity of a user*,* system*,* or entity attempting to access genomic data or computing resources*, typically using credentials such as passwords, biometric data, or security tokens. Effective, efficient and interoperable authentication supports responsible stewardship by permitting cloud-enabled access only to those users with bona fide research credentials. Consistent with reports from prior studies, authentication procedures vary across repository size, sponsor and project [9]. Standardizing authentication methods is a growing area of both technical [120] and policy efforts, with a focus on improving the interoperability of role-based access controls based on common credentials [121].

### Auditing

We defined auditing as the *process of systematically recording*,* reviewing*,* and analyzing access and usage logs to ensure proper handling of genomic data*,* support accountability*,* and detect security breaches or misuse*. Data misuse can still occur even if accessed by an authenticated, authorized user. Auditing data user permissions, activities and access controls can further reduce the risks of unauthorized access and noncompliant data use and therefore is an important feature of cloud-based data stewardship. Modern cloud platforms can also be more easily engineered with built-in auditing capabilities. Because researchers engage with data within a secure perimeter in the cloud, all activities can be monitored and logged for audit. In the post-data access phase, data access committees and other oversight bodies should perform periodic audits of researchers to verify accessed data are indeed used for approved purposes. Such auditing can also apply to data access and oversight decisions, providing users with information about the number of access requests, why requests were denied, or to enable review of the consistency of data access committee decisions as has been previously advocated [122–124].

### Security

We defined security as the cornerstone of safe data storage, encompassing *the practices and technologies implemented to protect genomic data from unauthorized access*,* corruption*,* or theft*, including encryption, firewalls, and intrusion detection systems. Re-identification of properly de-identified human genomic data also remains a concern for both researchers and data contributors alike. As such, security remained the most widely documented and highly supported indicator of responsible data stewardship practice in the literature we scoped. The individual outcomes relative to this domain highlight that security protocols are proportionate to the levels of protection required of the data hosted in the specific cloud database/repository and are based on the highest acceptable industry standards such as homomorphic encryption, multi-factor authentication and differential privacy. Security was also often referenced in relation to data quality. More secure data may be less vulnerable to data tampering and spoofing. This relationship was frequently cited in the literature and reflected in the outcomes that promote the security of all source data deposited in the cloud. Penalties and other sanctions for misuse of the data (e.g. re-identification) were proposed, as well as requirements to notify individuals of any breach or unauthorized access when possible.

### Sustainability

We defined sustainability as *the ability to maintain and support genomic data infrastructures*,* research programs*,* and access mechanisms over the long term*,* including financial*,* technical*,* and operational viability*. Significant human and material resources are needed to stand up a cloud-based database/repository, the maintenance of which optimizes data reusability. Sustaining existing cloud databases/repositories requires dedicated resources that may/not have been factored into original grant proposals. Once the platform has been established, data should be properly indexed and easily discoverable.

### Standardization

We defined standardization as *the process of ensuring that genomic data is collected*,* formatted*,* and annotated consistently*,* using common standards to promote interoperability*,* reuse*,* and accurate analysis*. Standardizing how data are collected and stored using standardized file formats expedites sharing and lowers barriers to reuse. Articles co-authored by large consortia, for example the Global Alliance for Genomics and Health [8], highlighted the need to adopt standardized formats and protocols that support data integration and cloud federation [125].

### Consent and Compliance

Consent and Compliance are considered jointly in this analysis due to their interdependence in data stewardship. We defined consent as *the normative boundaries of permissible data use*, whereas compliance *ensures these boundaries are systematically implemented and enforced through legal*,* ethical*,* and technical frameworks*. Respecting consent permissions, namely the informed, voluntary agreement by a research participant or patient for their genomic data to be collected, used, stored and potentially shared, with clear communication about purposes, risks and rights, should be prioritized in all phases of cloud data management, storage and sharing phases. Articles included in our review rarely discussed the actual consent process that preceded data collection and eventual deposit into a cloud database/repository. Rather, nearly every article discussed storing, analyzing and sharing only properly consented data. Individual core outcomes synthesized under this domain thus reflect this attention to verifying and preserving consent permissions in downstream data storage, use and sharing. Moreover, data contributors should be able to exercise their rights to data deletion or withdrawal of consent, whenever possible.

### Community Engagement

Engagement between data contributors and researcher communities offers more direct pathways for feedback and participation in data stewardship. We therefore defined community engagement as going *beyond the norms of individual consent to embrace collective ethical considerations*,* particularly in contexts where data contributions may affect community-level identities*,* rights*,* and interests*. Machine-readable consent permissions, automated authentication and role-based access controls, and model-to-data interactions in the cloud make it possible to engineer more granular data sharing choices than have been historically possible. Several articles discussed dynamic consent-enabling features of the cloud, and which can help overcome the limitations of broad consent. The improved accessibility to genomic data by leveraging cloud platforms can be both a benefit and hazard, particularly for data involving Indigenous [126] and other communities for whom community consultation and representation in data stewardship decisions is vital to data stewardship.

## Discussion

Our review shows that while data stewardship in the cloud has matured in many technical respects, it remains uneven across normative understanding of stewardship varies significantly across policy contexts.

In our analysis of the literature, responsible data stewardship takes a “life cycle” approach that is informed by a recordable provenance [127] of data sharing choices. Thus, it was crucial that our COS address all phases of the life cycle- from consent and collection to analysis and sharing. However, few articles contained the term “data stewardship,” indicating a general lack of consensus about how it is distinguished from related data governance concepts like privacy and protection, access and responsible use.

The proposed TA^3^S^3^C^2^ (pronounced “task”) core outcome set extends data stewardship responsibilities to cloud-based environments by drawing on the normative and practical recommendations from foundational frameworks for data sharing – namely FAIR, CARE, and TRUST. Because they are platform agnostic, existing frameworks may overlook meaningful opportunities for responsible stewardship in the cloud, specifically.

### Assumptions about the computing environment affect framework application

Foundational frameworks assume that data owners, data custodians, and repository managers control where their data resides and that the data sharing policies fall under jurisdiction-specific laws. However, cloud-based, global data networks allow for the sharing of data across jurisdictions, further complicating the issue of appropriate data use. For instance, CARE outlines the “Authority to Control” and “Responsibility.” The authority referenced in the principle “Authority to Control” relates both to data contributors (i.e. people who agree to have their data used) as well as the data users who control how data are stored and under what circumstances the data can be accessed.

Data may be stored in the cloud near where it was originally collected; it may be analyzed and accessed from data collection and possibly in jurisdictions with weaker data protections. In cloud-environments, governance responsibilities are also more widely distributed and easily fragmented across cloud service providers, repository managers, institutional leadership, and independent researchers, among others [10]. Prior efforts to guide responsible data stewardship have also taken for granted that humans will manually oversee all elements of the data management and responsible stewardship processes. Indeed, software and other machine-readable tools have been developed for the cloud to automate genomic data access and management decisions with notable success when compared to human-only decision-making (see for example the Data Use Ontology [69, 128, 129]). Accessibility in a global network may be affected by jurisdiction-based access restrictions, and findability may be dictated not by research need but by cloud provider-limited functionalities.

### Need for Objective Measures

Methods to develop outcome measures for genomic data stewardship are growing in popularity and need [130]. FAIR, CARE and TRUST provide strong foundations for what constitutes responsible data stewardship. Themes synthesized from this literature review around common data stewardship policies and practice has since activated a validation phase of this work. The development and later validation of the TA^3^S^3^C^2^ (pronounced “task”) core outcome set (see *Limitations*) is one viable methodological approach to ensuring data sharing policy and practice evolve at the pace of computing technologies. Distinguishing responsible data stewardship through objective measures provides a model for future applications as other repository architectures become more widely used. Conducting this scoping review greatly contributed to foundational knowledge about what cloud-based repositories should be measuring to optimize their responsible stewardship impact.

### Limitations

Our review indicates that data stewardship is highly contextual and conceptually fluid. In contrast to other data stewardship concepts with well-defined legal or regulatory meanings (e.g., data privacy), data stewardship lacks a consistent definition in the literature and is therefore interpreted in diverse ways.

To capture the core elements of data stewardship, we examined three key stages of the research data lifecycle: (1) pre-data collection, (2) data storage and use, and (3) secondary use and sharing. However, our scope may not encompass all data stewardship responsibilities as defined by specific institutional policy or applicable data protection laws everywhere in the world. We recognize, for example, that in European legal and policy discourse the related concept of the data trustee may foreground somewhat different expectations, particularly around fiduciary representation, data protection, and security. Future work could compare whether explicitly invoking these alternative conceptual frames changes the outputs generated.

Articles included in our review were predominantly authored by researchers in North America and Europe, where the largest commercial cloud service providers also reside (e.g. Amazon Web Services, Microsoft Azure and Google Cloud). Because the literature is oversampled from these regions, it largely focuses on the architectures and services of U.S.- and EU-based providers, potentially underrepresenting the experiences of researchers in other parts of the world who may rely on regional providers and face different constraints shaping data stewardship responsibilities. Future work will involve a three-round modified Delphi study with global experts in genomic data stewardship in which they will evaluate the importance and feasibility of the core outcome set. In this study, panelists will also suggest additional outcomes and categories that may not be present in our scoping review.

## Conclusion

The principle-to-practice translation of responsible data stewardship is an underdeveloped dimension of institutional data sharing practice in the wake of dataset migration to cloud platforms. Moving from research compliance to responsible stewardship is a normative goal that can be achieved through practical guidance for institutional data stewards. To bridge this translational gap, we synthesized an initial COS comprised of 9 core domains and 35 individual outcomes (TA^3^S^3^C^2^) from the literature. The proposed COS can support standard assessment of genomic data stewardship goals in service of sustaining trustworthy data infrastructures in the cloud. The scalability of cloud platforms for storing and co-locating genomic data analysis has the potential to reach more researchers with fewer access barriers than copy and download approaches alone. But as computing technologies underpinning data infrastructures evolve, new data stewardship opportunities and challenges will emerge that require bespoke guidance. The TA^3^S^3^C^2^ outcomes thus respond to an evolving need for branched frameworks that can translate data stewardship responsibilities to the specifications of new computing environments (e.g. cloud) and technologies (e.g. artificial intelligence).

Given the scoping nature of this review, future work will include refining the COS and developing corresponding criteria for assessing core outcomes among key informants, institutional data stewards and data curators alike. Validating the COS and assessment criteria across different cloud database/repository models and genomic research contexts will inform the future development of a data stewardship maturity matrix – conceptually similar to the Maturity Level Model for national genomics initiatives developed by the Beyond 1 Million Genomes Project [131] and Good Pharma Scorecard [132] – that repository managers and other institutional data stewards can use to determine whether, and how well their data stewardship practices compare relative to established maturity measures.

## Supplementary Information

Supplementary Material 1.

## Data Availability

Data extraction from 46 articles are available in **Supplementary Files 1**. Raw literature review data and records search can be available upon request.
