# Supplementary material for: Characterizing core outcomes of responsible stewardship for human genomic data in the cloud
Source: BMC Med Genomics. 2026 May 6;19:105. doi: 10.1186/s12920-026-02382-x (PMC13317215; doi:10.1186/s12920-026-02382-x)
Supplement: Supplementary file 1 — Supplementary Material 1. [file 12920_2026_2382_MOESM1_ESM.docx]

**Supplementary Materials 1.** Data extraction table of 46 included records in the review. The Reference number is the corresponding citation number.

| **Author** | **Ref #** | **Title** | **Year** | **Citation** |
| --- | --- | --- | --- | --- |
| Abbot E., Faunce, T. | ^89^ | Seeding Australian Regulation of Genomics in the Cloud | 2016 | Abbott, E. & Faunce, T. A. Seeding Australian Regulation of Genomics in the Cloud. SSRN Scholarly Paper at https://papers.ssrn.com/abstract=3125701 (2016). |
| Asiimwe, R. *et al.* | ^90^ | From biobank and data silos into a data commons: convergence to support translational medicine | 2021 | Asiimwe, R. *et al.* From biobank and data silos into a data commons: convergence to support translational medicine. *J. Transl. Med.* **19**, 493 (2021). |
| Aziz, M. *et al.* | ^91^ | Privacy-preserving techniques of genomic data- a survey | 2019 | Aziz, M. M. A. *et al.* Privacy-preserving techniques of genomic data—a survey. *Brief. Bioinform.* **20**, 887–895 (2019). |
| Bahmani, A. *et al.* | ^92^ | Swarm: A federated cloud framework for large-scale variant analysis | 2021 | Bahmani, A. *et al.* Swarm: A federated cloud framework for large-scale variant analysis. *PLOS Comput. Biol.* **17**, e1008977 (2021). |
| Calabrese, B. | ^93^ | Cloud-Based Bioinformatics Tools | 2019 | Calabrese, B. Cloud-Based Bioinformatics Tools. in *Encyclopedia of Bioinformatics and Computational Biology* (eds Ranganathan, S., Gribskov, M., Nakai, K. & Schönbach, C.) 252–256 (Academic Press, Oxford, 2019). doi:10.1016/B978-0-12-809633-8.20376-2. |
| Canakoglu, A. *et al.* | ^94^ | Federated sharing and processing of genomic datasets for tertiary data analysis | 2021 | Canakoglu, A. *et al.* Federated sharing and processing of genomic datasets for tertiary data analysis. *Brief. Bioinform.* **22**, bbaa091 (2021). |
| Carter, A. | ^95^ | Considerations for Genomic Data Privacy and Security when Working in the Cloud | 2019 | Carter, A. B. Considerations for Genomic Data Privacy and Security when Working in the Cloud. *J. Mol. Diagn.* **21**, 542–552 (2019). |
| Casaletto, J. *et al.* | ^96^ | Federated Analysis for Privacy-Preserving Data Sharing: A Technical and Legal Primer | 2023 | Casaletto, J., Bernier, A., McDougall, R. & Cline, M. S. Federated Analysis for Privacy-Preserving Data Sharing: A Technical and Legal Primer. *Annu. Rev. Genomics Hum. Genet.* **24**, 347–368 (2023). |
| Charlebois, K., Palmour, N., Knoppers, B. | ^12^ | The Adoption of Cloud Computing in the Field of Genomics Research: The Influence of Ethical and Legal Issues | 2016 | Charlebois, K., Palmour, N. & Knoppers, B. M. The Adoption of Cloud Computing in the Field of Genomics Research: The Influence of Ethical and Legal Issues. e0164347 (2016) doi:10.1371/journal.pone.0164347. |
| Church, P., Goscinski, A. | ^97^ | Selected Approaches and Frameworks to Carry out Genomic Data Analysis on the Cloud | 2015 | Church, P. C. & Goscinski, A. M. Selected Approaches and Frameworks to Carry Out Genomic Data Provision and Analysis on the Cloud. *Scalable Comput. Pract. Exp.* **16**, 19–35 (2015). |
| Conrad, L. *et al.* | ^98^ | Running Genomic Analyses in the Cloud | 2019 | Conrad, L. *et al.* Running Genomic Analyses in the Cloud. in *Digital Health: Changing the Way Healthcare is Conceptualised and Delivered* 149–155 (IOS Press, 2019). doi:10.3233/SHTI190787. |
| Dahlquist, J., Nelson, S., Fullerton, S. | ^99^ | Cloud-based biomedical data storage and analysis for genomic research: Landscape analysis of data governance in emerging NIH-supported platforms | 2023 | Dahlquist, J. M., Nelson, S. C. & Fullerton, S. M. Cloud-based biomedical data storage and analysis for genomic research: Landscape analysis of data governance in emerging NIH-supported platforms. *Hum. Genet. Genomics Adv.* **4**, (2023). |
| Dankar, F., Ptitsyn, A. | ^100^ | The development of large-scale de-identified biomedical databases in the age of genomics—principles and challenges | 2018 | Dankar, F. K., Ptitsyn, A. & Dankar, S. K. The development of large-scale de-identified biomedical databases in the age of genomics—principles and challenges. *Hum. Genomics* **12**, 19 (2018). |
| Datta, S., Bettinger, K., Snyder, M. | ^101^ | Secure cloud computing for genomic data | 2016 | Datta, S., Bettinger, K. & Snyder, M. Secure cloud computing for genomic data. *Nat. Biotechnol.* **34**, 588–591 (2016). |
| Deflaux, N. *et al.* | ^102^ | Demonstrating paths for unlocking the value of cloud genomics through cross cohort analysis | 2023 | Deflaux, N. *et al.* Demonstrating paths for unlocking the value of cloud genomics through cross cohort analysis. *Nat. Commun.* **14**, 5419 (2023). |
| Dove, E. *et al.* | ^13^ | Genomic cloud computing: legal and ethical points to consider | 2015 | Dove, E. S. *et al.* Genomic cloud computing: legal and ethical points to consider. *Eur. J. Hum. Genet.* **23**, 1271–1278 (2015). |
| Gholami, A., Dowling, J., Laure, E. | ^103^ | A security framework for population-scale genomics analysis | 2015 | Gholami, A., Dowling, J. & Laure, E. A security framework for population-scale genomics analysis. in *2015 International Conference on High Performance Computing & Simulation (HPCS)* 106–114 (IEEE, Amsterdam, Netherlands, 2015). doi:10.1109/HPCSim.2015.7237028. |
| Gim, J. | ^104^ | A Genomic Information Management System for Maintaining Healthy Genomic States and Application of Genomic Big Data in Clinical Research | 2022 | Gim, J.-A. A Genomic Information Management System for Maintaining Healthy Genomic States and Application of Genomic Big Data in Clinical Research. 5963 (2022) doi:10.3390/ijms23115963. |
| Granados Moreno, P., Joly, Y., Knoppers, B. | ^105^ | Public–Private Partnerships in Cloud-Computing Services in the Context of Genomic Research | 2017 | Granados Moreno, P., Joly, Y. & Knoppers, B. M. Public–Private Partnerships in Cloud-Computing Services in the Context of Genomic Research. *Front. Med.* **4**, (2017). |
| Greenbaum, D. *et al.* | ^106^ | Genomics and Privacy: Implications of the New Reality of Closed Data for the Field | 2011 | Greenbaum, D., Sboner, A., Mu, X. J. & Gerstein, M. Genomics and Privacy: Implications of the New Reality of Closed Data for the Field. *PLoS Comput. Biol.* **7**, e1002278 (2011). |
| Greenbaum, D., Gerstein, M. | ^14^ | The Role of Cloud Computing in Managing the Deluge of Potentially Private Genetic Data | 2011 | Greenbaum, D. & Gerstein, M. The Role of Cloud Computing in Managing the Deluge of Potentially Private Genetic Data. *Am. J. Bioeth.* **11**, 39–41 (2011). |
| Grossman, R. | ^107^ | Data Lakes, Clouds, and Commons: A Review of Platforms for Analyzing and Sharing Genomic Data | 2019 | Grossman, R. L. Data Lakes, Clouds, and Commons: A Review of Platforms for Analyzing and Sharing Genomic Data. *Trends Genet.* **35**, 223–234 (2019). |
| Groth, P., Reuter, G., Thieme, S. | ^108^ | Analysis of Genomic Data in a Cloud Computing Environment | 2015 | Groth, P., Reuter, G. & Thieme, S. Analysis of Genomic Data in a Cloud Computing Environment. in *Big Data Analytics in Bioinformatics and Healthcare* 186–214 (IGI Global, 2015). doi:10.4018/978-1-4666-6611-5.ch009. |
| Heath, A. *et al.* | ^109^ | Bionimbus: a cloud for managing, analyzing and sharing large genomics datasets | 2014 | Heath, A. P. *et al.* Bionimbus: a cloud for managing, analyzing and sharing large genomics datasets. *J. Am. Med. Inform. Assoc.* **21**, 969–975 (2014). |
| Jafarbeiki, S. *et al.* | ^110^ | Collaborative analysis of genomic data: vision and challenges | 2022 | Jafarbeiki, S., Gaire, R., Sakzad, A., Kermanshahi, S. K. & Steinfeld, R. Collaborative analysis of genomic data: vision and challenges. Preprint at http://arxiv.org/abs/2202.04841 (2022). |
| Krampis, K., Wultsch, C. | ^111^ | A Review of Cloud Computing Bioinformatics Solutions for Next-Gen Sequencing Data Analysis and Research | 2015 | Krampis, K. & Wultsch, C. A Review of Cloud Computing Bioinformatics Solutions for Next-Gen Sequencing Data Analysis and Research. *Methods Gener. Seq.* **2**, (2015). |
| Kusunose, M., Muto, K. | ^112^ | Public attitudes toward cloud computing and willingness to share personal health records (PHRs) and genome data for health care research in Japan | 2023 | Kusunose, M. & Muto, K. Public attitudes toward cloud computing and willingness to share personal health records (PHRs) and genome data for health care research in Japan. *Hum. Genome Var.* **10**, (2023). |
| Langmead, B., Nellore, A. | ^113^ | Cloud computing for genomic data analysis and collaboration | 2018 | Langmead, B. & Nellore, A. Cloud computing for genomic data analysis and collaboration. *Nat. Rev. Genet.* **19**, 208–219 (2018). |
| Lau, J. *et al.* | ^114^ | The Cancer Genomics Cloud: Collaborative, Reproducible, and Democratized—A New Paradigm in Large-Scale Computational Research | 2017 | Lau, J. W. *et al.* The Cancer Genomics Cloud: Collaborative, Reproducible, and Democratized—A New Paradigm in Large-Scale Computational Research. *Cancer Res.* **77**, e3–e6 (2017). |
| McLeod, C. *et al.* | ^115^ | St. Jude Cloud—a Pediatric Cancer Genomic Data Sharing Ecosystem | 2021 | McLeod, C. *et al.* St. Jude Cloud: A Pediatric Cancer Genomic Data-Sharing Ecosystem. *Cancer Discov.* **11**, 1082–1099 (2021). |
| Navale, V., McAuliffe. | ^116^ | Long-term preservation of biomedical research data | 2018 | Navale, V. & McAuliffe, M. Long-term preservation of biomedical research data. *F1000Research* **7**, 1353 (2018). |
| Ohno-Machado, L. *et al.* | ^117^ | Sharing data for the public good and protecting individual privacy: informatics solutions to combine different goals | 2013 | Ohno-Machado, L. Sharing data for the public good and protecting individual privacy: informatics solutions to combine different goals. *J. Am. Med. Inform. Assoc.* **20**, 1–1 (2013). |
| Phillips, M., Knoppers, B., Joly, Y. | ^118^ | Seeking a "Race to the Top" in Genomic Cloud Privacy? | 2015 | Mark Phillips, B. Knoppers, & Y. Joly. Seeking a ‘Race to the Top’ in Genomic Cloud Privacy? *2015 IEEE Secur. Priv. Workshop* (2015). |
| Prasanna, A. *et al.* | ^119^ | Cloud Based Solutions for Genome Informatics: Challenges and Applications | 2018 | Prasanna, A., Pooja, R., Suchithra, V., Ravikumar, A. & Niranjan, V. Cloud Based Solutions for Genome Informatics: Challenges and Applications. in vol. 5 10652–10659 (Elsevier Ltd, 2018). |
| Reynolds, S. *et al.* | ^120^ | The ISB Cancer Genomics Cloud: A Flexible Cloud-based Platform for Cancer Genomics Research | 2017 | Reynolds, S. M. *et al.* The ISB Cancer Genomics Cloud: A Flexible Cloud-based Platform for Cancer Genomics Research. *Cancer Res.* **77**, e7–e10 (2017). |
| Rilak, Z., Wernicke, S., Bogicevic, I. | ^121^ | Keeping Genomic Data Safe on the Cloud | 2014 | Rilak, Z., Wernicke, S. & Bogicevic, I. Keeping Genomic Data Safe on the Cloud. *J. Biomol. Tech.* **25**, S5–S5 (2014). |
| Schatz, M. *et al*. | ^122^ | Inverting the model of genomics data sharing with the NHGRI Genomic Data Science Analysis, Visualization, and Informatics Lab-space | 2022 | Schatz, M. C. *et al.* Inverting the model of genomics data sharing with the NHGRI Genomic Data Science Analysis, Visualization, and Informatics Lab-space. *Cell Genomics* **2**, 100085 (2022). |
| Schlosberg, A. | ^123^ | Data security in genomics: A review of Australian privacy requirements and their relation to cryptography in data storage | 2016 | Schlosberg, A. Data security in genomics: A review of Australian privacy requirements and their relation to cryptography in data storage. *J. Pathol. Inform.* **7**, 6 (2016). |
| Shih, C. *et al*. | ^124^ | A five-safes approach to a secure and scalable genomics data repository | 2023 | Shih, C. C. *et al.* A five-safes approach to a secure and scalable genomics data repository. *iScience* **26**, 106546 (2023). |
| Sousa, J. *et al*. | ^125^ | Efficient and secure outsourcing of genomic data storage | 2017 | Sousa, J. S. *et al.* Efficient and secure outsourcing of genomic data storage. *BMC Med. Genomics* **10**, 46 (2017). |
| Stein, L. *et al.* | ^126^ | Data analysis: Create a cloud commons | 2015 | Stein, L. D., Knoppers, B. M., Campbell, P., Getz, G. & Korbel, J. O. Data analysis: Create a cloud commons. *Nat. Lond.* **523**, 149–151 (2015). |
| Tang, H. *et al*. | ^127^ | Protecting genomic data analytics in the cloud: state of the art and opportunities | 2016 | Tang, H. *et al.* Protecting genomic data analytics in the cloud: state of the art and opportunities. *BMC Med. Genomics* **9**, 63 (2016). |
| The Global Alliance for Genomics and Health | ^128^ | A federated ecosystem for sharing genomic, clinical data | 2016 | The Global Alliance for Genomics and Health. A federated ecosystem for sharing genomic, clinical data. *Science* **352**, 1278–1280 (2016). |
| Thorogood, A. *et al.* | ^129^ | Protecting the Privacy of Canadians' Health Information in the Cloud | 2016 | Thorogood, A., Simkevitz, H., Phillips, M., Dove, E. & Joly, Y. Protecting the Privacy of Canadians’ Health Information in the Cloud. *Can. J. Law Technol.* **14**, (2016). |
| Via, M. | ^130^ | Big Data in Genomics: Ethical Challenges and Risks | 2024 | Via, M. Big Data in Genomics: Ethical Challenges and Risks. *Rev. Bioét. Derecho* 33–45 (2024). |
| Warth, B. *et al.* | ^131^ | Metabolizing Data in the Cloud | 2017 | Warth, B. *et al.* Metabolizing Data in the Cloud. *Trends Biotechnol.* **35**, 481–483 (2017). |
